# Thyroid metastasis from hepatocellular carcinoma: a rare case report and literature review

**DOI:** 10.3389/fonc.2025.1581927

**Published:** 2025-06-12

**Authors:** Shasha Guan, Qianwen Ye, Panhua Li, Lijuan Ding

**Affiliations:** Oncology Department, Hainan Hospital of Chinese People’s Liberation Army (PLA) General Hospital, Sanya, China

**Keywords:** hepatocellular carcinoma, liver cancer, thyroid metastasis, transcatheter arterial chemotherapy embolization (TACE), case report

## Abstract

**Background:**

Thyroid metastasis is a relatively uncommon event in clinical practice, typically occurs in more prevalent primary tumors, including renal cell carcinoma and cancers of the gastrointestinal tract, lungs, and breast. The incidence of thyroid metastasis from primary hepatocellular carcinoma (HCC) is particularly infrequent. This case report outlines the clinical challenges and diagnostic pathway associated with a thyroid mass in a patient with HCC, highlighting the rarity and intricacy of such metastatic associations.

**Case Presentation:**

A 51-year-old male with a long-standing history of hepatitis B-related liver cirrhosis presented with a rapidly enlarging painful left-sided thyroid mass 28 months after a diagnosis of HCC. FDG-PET/CT imaging revealed a 6 cm hypodense tumor in the left lobe of the thyroid and further fine needle aspiration cytology and biopsy (FNAB) confirmed it as a metastasis from HCC. The patient underwent transcatheter arterial chemotherapy embolization (TACE) as a first attempt to control the progress of the thyroid lesion. However, subsequent imaging showed a continued progression of the lesions in liver, along with other metastatic sites. Although multiple interventions, such as radiofrequency ablation (RFA) procedures was administered due to the progression of liver cancer and embolization therapies for the thyroid, the patient experienced significant deterioration, presenting with respiratory failure due to malignant pleural effusion.

**Conclusions:**

This unique case highlights the importance of promptly considering persistent or newly developed thyroid nodules as indicative of metastatic disease. The identification of thyroid metastases, especially in the context of extensive organ involvement, often correlates with a poor prognosis. Given the distinctive physiological properties of the thyroid gland, multidisciplinary management may offer clinical benefits for such patients with complex metastatic profiles. Combining locoregional therapies with immunotherapy, particularly dual immunotherapy, may offer significant prognostic advantages. Nevertheless, these hypotheses need to be verified through large-scale clinical trials.

## Introduction

The thyroid gland, due to its unique anatomical and physiological properties, is rarely involved in metastatic disease. It was once believed that the gland’s high blood supply and iodine content prevented the establishment of metastatic foci. However, contemporary studies reveal that thyroid metastases occur in approximately 1.4% to 3% of all thyroid malignancies, while autopsy findings show a wider range of incidence from 1.9% to 24% (1). This discrepancy indicates an underestimation of metastatic thyroid cancer in clinical practice. Renal cell carcinoma is the primary source of metastases to the thyroid, accounting for nearly 48%, yet hepatocellular carcinoma (HCC) emerges infrequently in these reports, with a noted incidence of around 2% (2).

The occurrence of HCC metastasizing to the thyroid gland represents not only a diagnostic rarity but also signifies a more advanced and aggressive stage of the disease. This highlights the critical need to thoroughly evaluate thyroid lesions in patients with primary liver cancer, especially those who have chronic viral hepatitis and cirrhosis (3). This case report aims to illuminate this uncommon phenomenon by detailing the diagnostic process involved, thereby enriching the limited existing literature pertaining to atypical metastatic manifestations of HCC.

## Patient information

**Figure 1:**
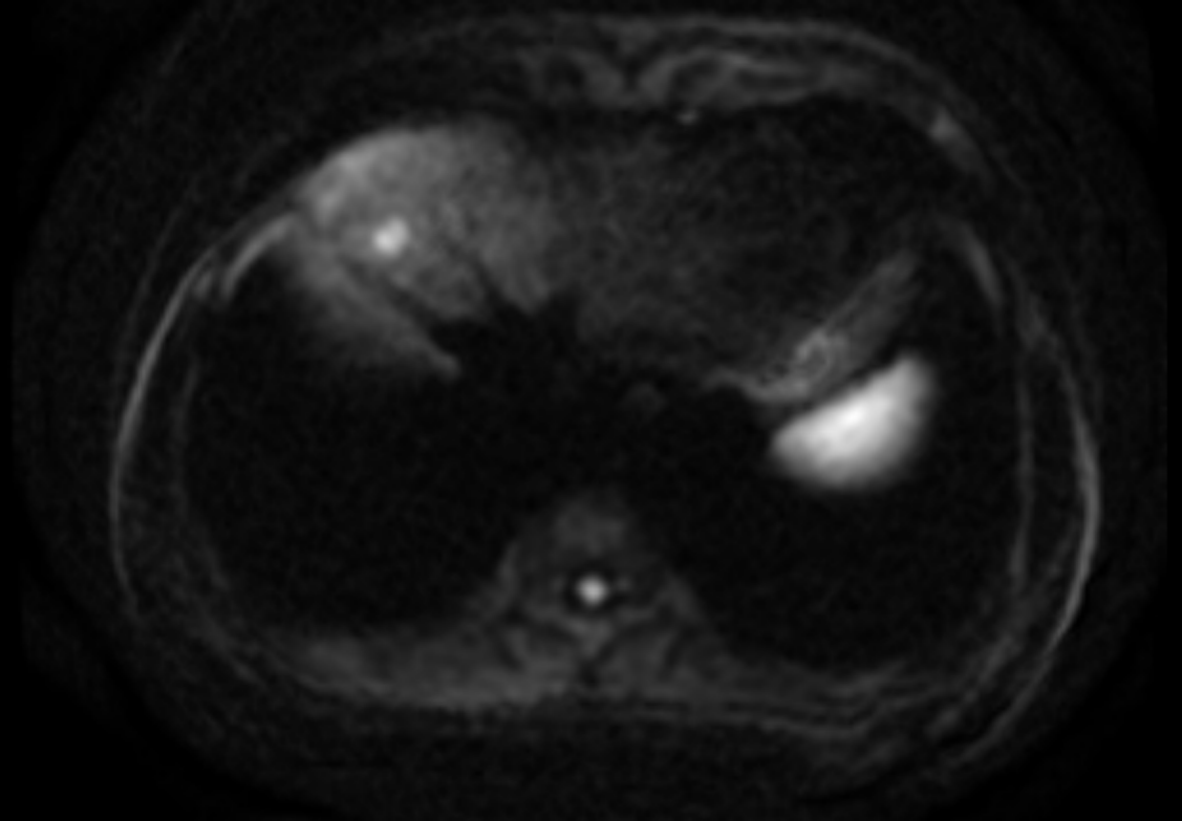
Enhanced abdominal MRI indicated a small nodule in the anterior segment of the right lobe of the liver, consistent with primary hepatocellular carcinoma.

A 49-year-old Han Chinese male, diagnosed with hepatitis B in 1997 and treated with one daily tablet of entecavir, visited our clinic in December 2014. His tests showed elevated serum α-fetoprotein levels of 80.5 ng/ml, well above the normal range of 0–20 ng/ml, and liver function abnormalities, with alanine aminotransferase (ALT) levels at 170 U/L (normal: 0–40 U/L) and aspartate aminotransferase (AST) levels at 111 U/L (normal: 0–40 U/L). A thorough abdominal MRI demonstrated a small 1.1cm lesion in the right lobe of the liver, leading to the diagnosis of primary HCC (**Figure 1**). The patient underwent radiofrequency ablation (RFA) of the tumor in January 2015, followed by multiple RFA therapies due to recurrence.

**Figure 2:**
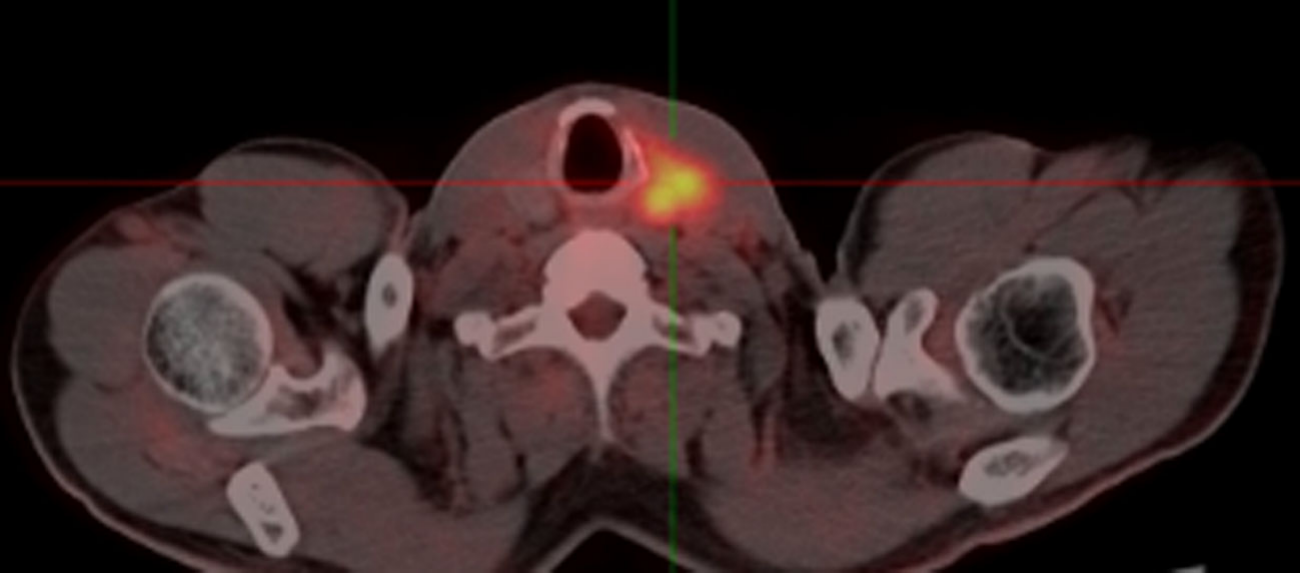
The FDG-PET/CT showing a highly metabolic focus in the the left lobe of the thyroid. FDG-PET/CT, fluorodeoxyglucose positron emission tomography/computed tomography.

**Figure 3:**
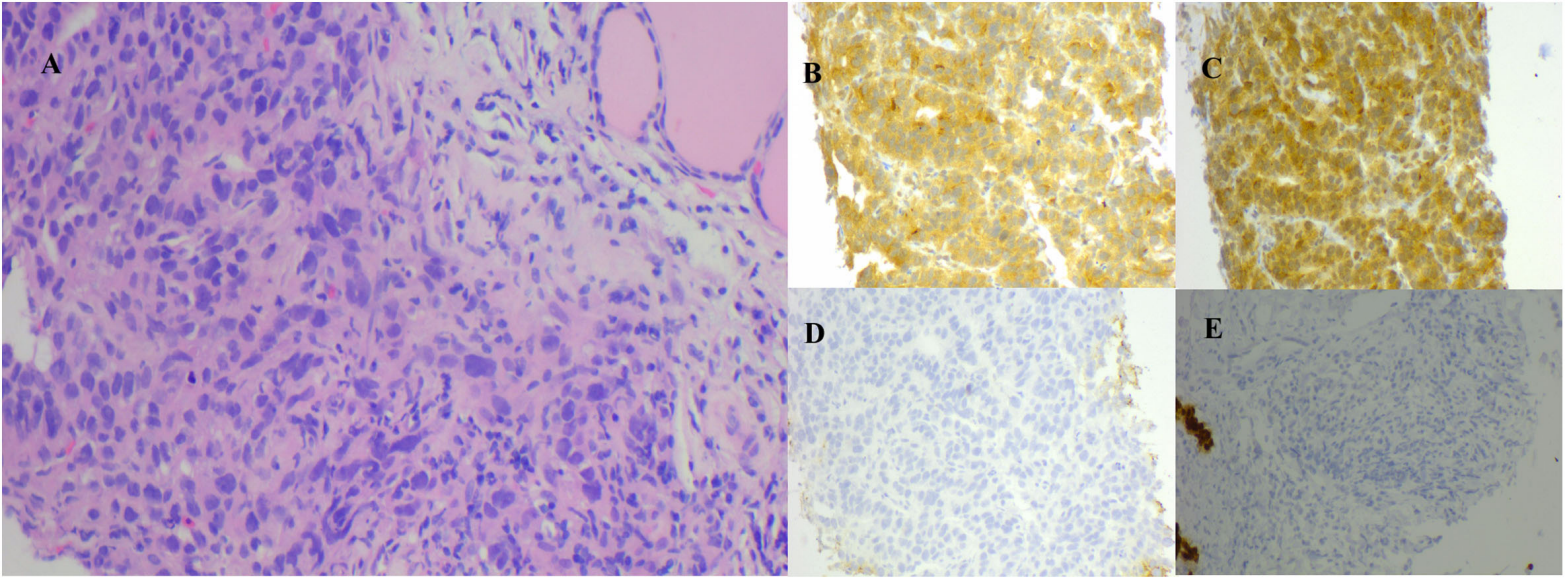
**(A)** FNAB of the thyroid, post HE, corroborates the pathology of hepatocellular carcinoma metastasized to the thyroid. Immunohistochemical examination was positive for AFP **(B)** and GPC3 **(C)**, and negative for TG **(D)** and TTF-1 **(E)** (HE magnification of 200x). AFP, alpha-fetoprotein; FNAB, fine needle aspiration biopsy; GPC3, glypican-3; HE, hematoxylin and eosin staining; TG, thyroglobulin; TTF-1, thyroid transcription factor-1.

The patient’s condition remained stable until April 2017 when he noticed a palpable, soft mass in the left lobe of his thyroid, measuring approximately 6 cm. A fluorodeoxyglucose positron emission tomography/computed tomography (FDG-PET/CT) was performed, which revealed a hypodense neoplasm located in the left lobe of the thyroid. This tumor demonstrated significantly elevated uptake of FDG, achieving a maximum standardized uptake value (SUV) of 8.5 (**Figure 2**). Metastasis from HCC was suspected. Fine needle aspiration cytology and biopsy (FNAB) revealed poorly differentiated carcinoma infiltrating the thyroid tissue, and immunohistochemical staining indicated features consistent with metastasis from HCC. Notably, the immunohistochemical profile showed positivity for glypican-3 (GPC3) and alpha-fetoprotein (AFP), while thyroid transcription factor-1 (TTF-1), thyroglobulin (TG) and hepatocyte markers were negative (**Figure 3**).

**Figure 4:**
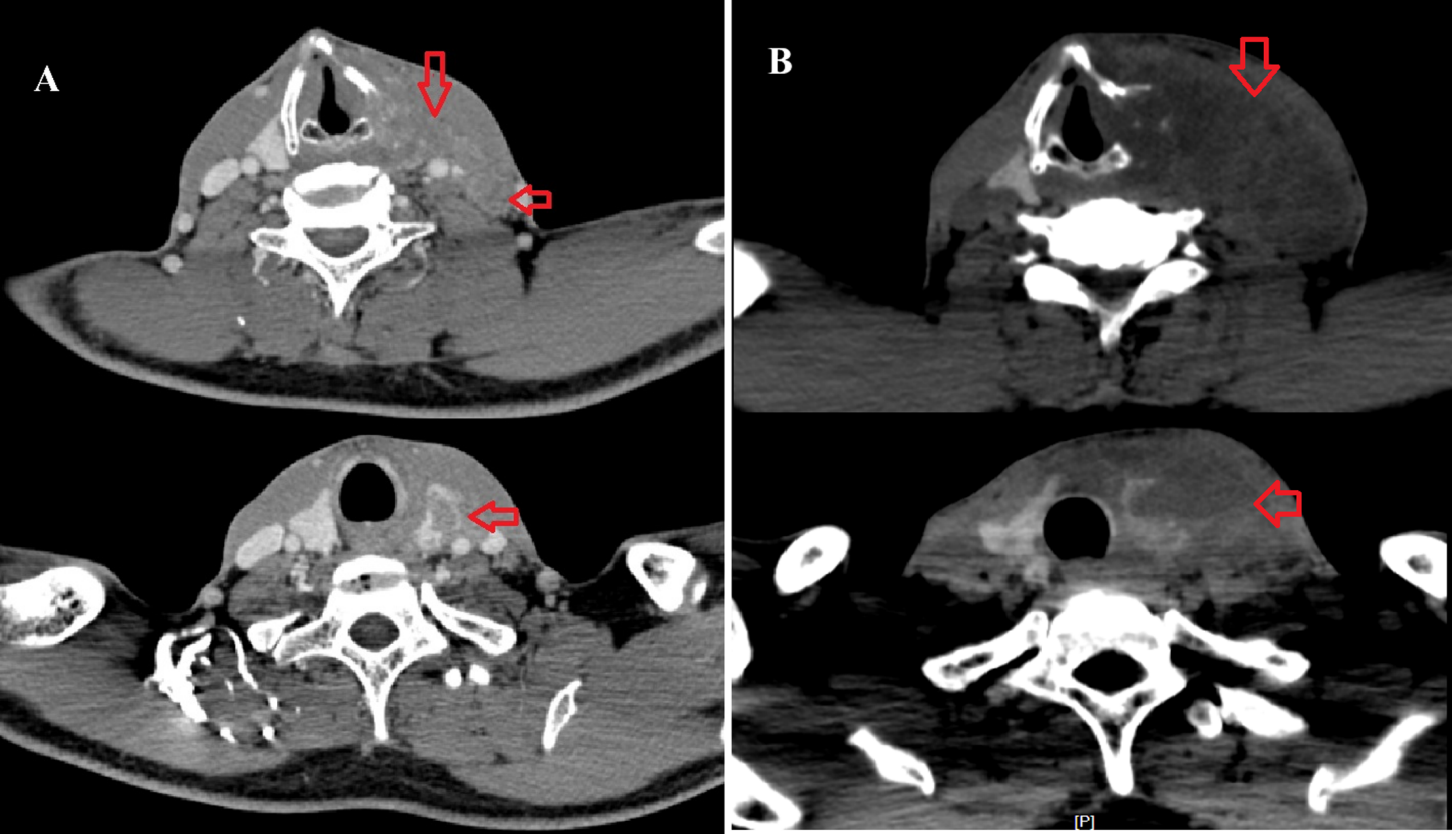
**(A)** A cervical enhanced CT scan on June 2017 indicated an irregular mass in the left thyroid lobe and enlarged lymph nodes in the left carotid sheath (arrow). **(B)** A cervical enhanced CT scan on November 2017 indicated an irregular mass in the left thyroid lobe consistent with metastasis, with adjacent destruction of the thyroid cartilage, involvement of the left hypopharynx, and possible thrombosis of the left internal jugular vein.

In June 2017, imaging revealed extensive disease progression in the liver and thyroid. A neck CT scan demonstrated malignant thyroid lesions infiltrating surrounding structures, including the esophagus and retropharyngeal space (**Figure 4A**). Additionally, bilateral tumor thrombi were noted in the internal jugular veins, indicative of aggressive metastatic disease. On June 29, 2017, hepatic artery angiography and hepatic transcatheter arterial chemotherapy embolization (TACE) were performed, during which 50 mg of oxaliplatin and 20 mg of epirubicin were administered. Due to the continuous progression of liver lesions and extensive involvement of the thyroid lesion, no radical surgery was performed on the thyroid metastasis.

**Figure 5:**
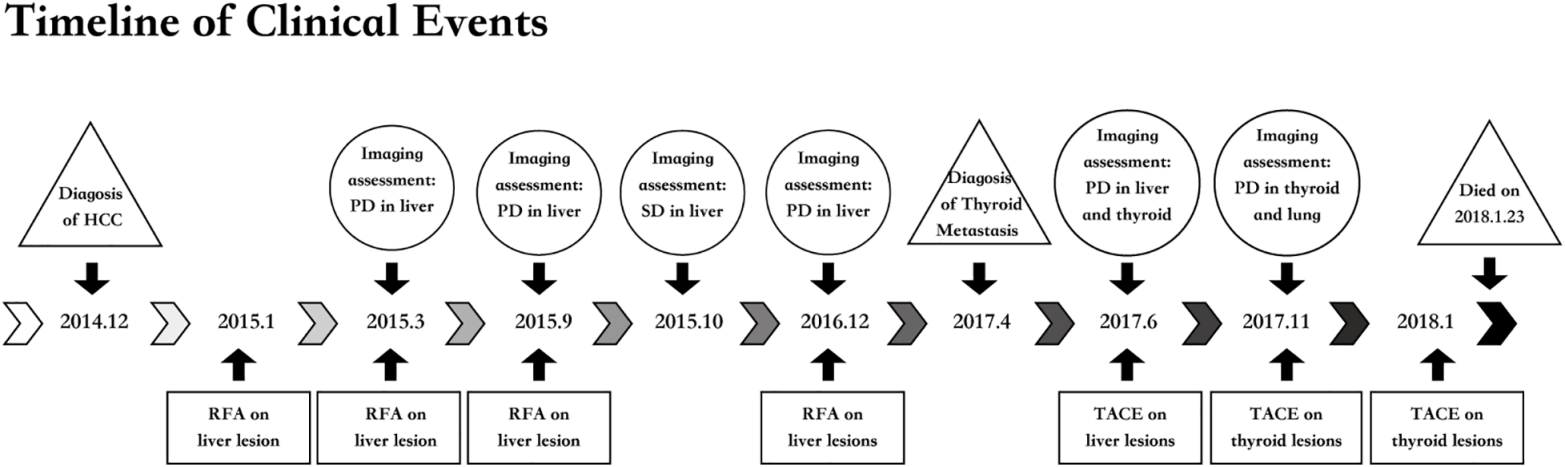
Timeline of clinical rvents. HCC, hepatocellular carcinoma; PD, progression disease; RFA, radiofrequency ablation; SD, stable disease; TACE, transcatheter arterial chemotherapy embolization.

A follow-up cervical CT on November 21, 2017, indicated an irregular mass in the left thyroid lobe consistent with metastasis. The mass showed invasion into the thyroid cartilage, affected the left hypopharynx, and may obstruct the left internal jugular vein (**Figure 4B**). Enlarged lymph nodes in the left cervical region were also noted. Multiple bilateral lung nodules were confirmed at the same time. Artery angiography and TACE were applied on the thyroid metastasis, during which 30 mg of lobaplatin, 250 mg of fluorouracil, and 10 mg of epirubicin were administered. Despite empirical interventions, including chemotherapeutic embolization to manage his thyroid lesions, the patient’s condition deteriorated markedly. A pleural effusion test revealed bloody fluid with cytologic findings consistent with hepatocellular carcinoma cells. Consequently, the patient succumbed to pulmonary failure secondary to extensive pleural and pulmonary metastasis on January 23, 2018. The overall survival (OS) was 36 months. All clinical events of this case was outlined in a timeline as shown in **Figure 5**.

## Discussion

HCC is one of the leading causes of cancer-associated mortality globally, characterized by its aggressive behavior and propensity for vascular invasion (4). In our case, the patient, a 51-year-old male diagnosed with primary liver cancer over 28 months ago, presented with thyroid metastasis originating from HCC. This case highlights the unusual yet critical phenomenon of intrathyroidal metastasis from an otherwise prevalent malignancy, which is scarcely documented in the medical literature. Typically, the metastatic characteristics of HCC is primarily marked by its tendency to disseminate to the lungs, bones, adrenal glands, and, less frequently, the brain. Such dissemination patterns are driven by the liver’s vascular architecture and the venous drainage pathways that have direct links to these specific organs. However, the occurrence of metastasis to the thyroid gland is extremely uncommon, with only a limited number of cases reported in the existing medical literature. A total of 11 cases have been reported in the literatures (2, 3, 5–13). In these cases, only one case occurred concurrently with HCC, whereas the other ten cases presented at different times, resulting in an average delay of 20 months. The clinical manifestations of thyroid metastases included a palpable thyroid mass, which can resemble both benign and malignant thyroid disorders. Of the 11 cases, just one displayed widespread involvement throughout the entire thyroid gland, whereas the remaining ten demonstrated localized nodules within the thyroid tissue. This may lead to diagnostic delays, as thyroid metastasis often presents with asymptomatic low-volume disease or atypical symptoms, such as dysphagia and hoarseness. Similarly, our patient exhibited simple symptoms correlated with primary thyroid disease. These symptoms underscore the necessity for heightened awareness of thyroid metastasis in patients with a history of HCC, particularly when new nodules arise.

Regarding the metastatic mechanisms, Willis put forth two hypotheses in 1931 to account for the infrequent occurrence of secondary thyroid tumors. First, the rapid arterial blood circulation in the thyroid hinders malignant cell adhesion. Second, the high oxygen saturation and iodine concentration in the thyroid suppress the proliferation of malignant tumor cells (1, 14–16). At present, it is also believed that poor or interrupted blood supply or decreased concentrations of iodine and oxygen are beneficial to the deposition of tumor cells (17–19). Nonetheless, a recent investigation indicates that the abundant blood supply of the thyroid may be a contributing factor to its susceptibility to metastatic cancer (16). Currently, there is no consensus on mechanism, and further in-depth research is required to investigate this issue.

Due to the clinical manifestation of metastatic thyroid tumor being hidden and lacking specificity, it is crucial to differentiate these cases from primary thyroid neoplasms, benign thyroid nodules, locally invasive conditions affecting the larynx or esophagus that encroach upon the thyroid, as well as various other pathologies. In contrast to primary thyroid tumors, metastases to the thyroid gland exhibit interstitial infiltration and lead to the encasement of follicles by tumor cells. The neck CT scan of our patient shows an irregular focus with destruction of adjacent structures, indicating metastasis. These findings, combined with immunohistochemistry (IHC), are crucial for distinguishing primary thyroid tumors from metastatic ones (1). Thyroid transcription factor-1 (TTF-1), classified within the NKx2 family of homeodomain transcription factors, functions as a key regulator in the tissue-specific transcription of the TG gene in the thyroid gland (20). TTF-1 is expressed in the thyroid follicle, parathyroid gland, alveolar epithelium, and diencephalon, highlighting its significant application utility in the clinical differentiation of thyroid or pulmonary neoplasms. It is reported that TTF-1 and TG are demonstrable through immunohistochemical methods in approximately 75% of thyroid tumors. When contrasted with TG, an antibody to TTF-1 is a more sensitive marker for poorly differentiated carcinomas and metastasis (21). TTF-1 is found to be expressed in approximately 75% of non-mucinous lung adenocarcinomas. Furthermore, the positive ratio of TTF-1 is significantly associated with the degree of differentiation within adenocarcinoma tissues (20). The elevation of AFP is invovled in several physiological and pathological conditions, including hepatocyte regeneration, hepatocarcinogenesis, and embryonic carcinomas. Notably, a increase in AFP levels exceeding 500 ng/ml has been linked with tumor size. Approximately 80% of small HCC cases do not exhibit any significant rise in AFP concentrations (22). The sensitivity of AFP, ranging from 25% to 65%, is unsatisfactory for the diagnosis of HCC, resulting in false negative outcomes in 50% of instances, particularly when identifying early-stage HCC. GPC3 is one of the members of heparan sulphate proteoglycans. It is involved in the modulation of cell growth and survival during embryonic development, primarily by influencing the activity of a range of growth factors. Additionally, it functions as a tumor suppressor. Research indicates that GPC3 expression is elevated in patients with HCC while it remains undetectable in healthy subjects and in those suffering from benign hepatic diseases, including dysplastic or cirrhotic nodules (23). Serum GPC3 outperforms AFP as a sensitive marker for early-stage HCC. A recent study assessed the diagnostic utility of GPC3 in combination with of AFP for HCC, showing a specificity and sensitivity of the two indices of 0.925 and 0.881, respectively, compared with 0.879 and 0.795 GPC3 alone (24). Histologically, the left thyroid lobe biopsy of our patient presents negative markers for primary thyroid neoplasms (TTF-1 and TG) and positive markers for hepatitis-associated disease (AFP and GPC3), confirming the presence of poorly differentiated metastatic HCC. The results emphasized the need for thorough immunohistochemical profiling.

The management of secondary thyroid tumors primarily encompasses surgery, radiotherapy, and chemotherapy. Nonetheless, there remains ongoing debate regarding the necessity of surgical intervention for patients with metastatic involvement of the thyroid gland. Approximately 35% to 80% of thyroid metastatic cancer patients experience the spread of the disease to multiple organs, resulting in a diminished prognosis. A systematic review including 147 cases of thyroid metastases from renal cell carcinoma indicates no significant difference in outcomes between patients who underwent surgery and those who did not. At one-year follow-up, the survival rate among patients who underwent surgical intervention was 55.6%, in comparison to a survival rate of 35.3% for those who did not receive surgery, though this was not statistically significant (P=0.1) (25). However, Russell et al. pointed out that patients who have undergone thyroidectomy survive longer, especially those with metastatic renal cell carcinoma (RCC). They also found that the longer survival time of patients is related to the longer interval between the diagnosis of the primary tumor and the occurrence of metastases to the thyroid gland (26). In colorectal cancer and lung cancer, the overall survival rate of patients with thyroid metastasis is lower because of the stronger invasiveness of their primary tumors (27). Thus, when the primary tumor has stronger inertia (such as kidney and breast tumors), the survival rate of patients is higher. Compared with primary thyroid tumors, secondary thyroid neoplasms are less sensitive to radioactive iodine (15). Therefore, for patients who cannot undergo surgical treatment, radiotherapy or chemotherapy is feasible, but the effect is not ideal (26, 28). Unfortunately, our cases did not have access to therapeutic interventions because of the large tumor and advanced disease progression, as shown in **Figure 4**. The angiography and transarterial chemoembolization (TACE) were performed on the thyroid lesion, but the effect was not satisfactory. This represents our first attempt at TACE for thyroid disease. It is documented that transcatheter arterial embolization (TAE) was applied in thyroid diseases only for hemostatic treatment (29–31). However, no reports on TACE for thyroid neoplasms could be reached on PubMed. The suboptimal therapeutic effect may result from the large tumor size (6 cm at diagnosis) and the rapid progression of the overall tumor burden. Furthermore, the implementation of TACE occurred 7 months after the discovery of the thyroid tumor, resulting in a delay in receiving potentially more effective treatment.

It is unfortunate that our patient failed to receive chemotherapy or sorafenib throughout the entire course. The management of advanced or unresectable HCC has evolved remarkably in recent years, primarily due to the emergence of targeted therapies and immune checkpoint inhibitors. The IMbrave150 (32) and ORIENT-32 (33) trials have significantly advanced the treatment landscape for advanced HCC by demonstrating the effectiveness of combined immune checkpoint inhibitors and anti-vascular therapies, showing the improved median OS of 19.2 months and 16.2 months, and the impressive disease control rate (DCR) of 56.5% and 70.6%, respectively. These results highlight the synergistic potential of targeting both immune and vascular pathways, leading to improved survival outcomes and disease management. Additionally, the HIMALAYA trial (34, 35) and Checkmate-9DW have made significant contributions to the realm of dual immunotherapy in first-line treatment for advanced HCC. The CARES-310 trial (36) marks a significant milestone by investigating the combination of lenvatinib, an oral multikinase inhibitor, with immune checkpoint inhibitors in advanced HCC. The findings indicated that this novel treatment strategy can achieve a median OS of 19.0 months and a DCR of 68.3%. The integration of immune checkpoint inhibitors with small molecule kinase inhibitors or anti-angiogenic agents is paving the way for a new treatment paradigm. This approach highlights the need for personalized treatment strategies that improve therapeutic outcomes and offer better disease control for patients. For our patient, TACE combined with immunotherapy might also serve as a more effective option. On one hand, the predominant etiology of HCC is the hepatitis B virus for this patient, which could potentially respond more favorably to immunotherapy (37). On the other hand, the efficacy of TACE combined with chemotherapy or immunotherapy remains unclear. Multiple randomized controlled trials that investigated the combination of TACE with systemic therapies did not demonstrate a significant improvement in OS. However, the TACTICS trial reported a notable enhancement in median progression-free survival (PFS) of approximately 12 months when TACE was administered alongside sorafenib compared to TACE as a standalone treatment (38–41). A study that investigated the efficacy of TACE plus camrelizumab and apatinib for patients with HCC in a real-world setting demonstrated significantly better OS, PFS, and objective response rate in the combination group (42). TACE could upregulate the expression of PD-1 and PD-L1 in HCC. Additionally, it could trigger apoptosis in tumor cells with the liberation of tumor-associated antigens, proinflammatory cytokines, vascular endothelial growth factor, and hypoxia-inducible factor 1-alpha (43–45). Such effects could convert an immunosuppressive “cold tumor” into an immunostimulatory “hot tumor”, which may enhance the immune response to immune checkpoint inhibitors. Nevertheless, the deeper mechanisms still require further research, and the survival benefit needs to be evaluated and validated in a subsequent large-scale randomized controlled trial.

## Conclusion

In conclusion, this case advocates for a vigilant approach towards the surveillance of thyroid lesions in patients with known HCC. In clinical practice, we suggest that prompt FNAB and immunohistochemical assessment are crucial. When treating patients with metastases to the thyroid gland, it is essential to comprehensively consider the characteristics and metastasis of the primary tumor, as well as the patient’s tolerance to surgery and life expectancy. The evidence presented in this report demonstrates the challenges associated with diagnosing intrathyroidal metastases and treating large intrathyroidal metastases. It calls for further research to delineate the mechanisms governing such metastatic processes, facilitating more effective management strategies for afflicted patients.

## Data Availability

The original contributions presented in the study are included in the article/supplementary material. Further inquiries can be directed to the corresponding author.
